# Establishment of a Murine Pro-acinar Cell Line to Characterize Roles for FGF2 and α3β1 Integrins in Regulating Pro-acinar Characteristics

**DOI:** 10.1038/s41598-019-47387-y

**Published:** 2019-07-29

**Authors:** Renée F. Thiemann, Deirdre A. Nelson, C. Michael DiPersio, Melinda Larsen, Susan E. LaFlamme

**Affiliations:** 1 Department of Regenerative & Cancer Cell Biology, Albany Medical College, 47 New Scotland Avenue, Albany, NY 12208 USA; 2 Department of Biological Sciences, University at Albany, State University of New York, 1400 Washington Avenue, Albany, NY 12222 USA; 3 Department of Surgery, Albany Medical Center, 43 New Scotland Avenue, Albany, NY 12208 USA

**Keywords:** Integrins, Molecular medicine

## Abstract

Radiation therapy for head and neck cancers results in permanent damage to the saliva producing acinar compartment of the salivary gland. To date, a pure pro-acinar cell line to study underlying mechanisms of acinar cell differentiation in culture has not been described. Here, we report the establishment of a pro-acinar (mSG-PAC1) and ductal (mSG-DUC1) cell line, from the murine submandibular salivary gland (SMG), which recapitulate developmental milestones in differentiation. mSG-DUC1 cells express the ductal markers, keratin-7 and keratin-19, and form lumenized spheroids. mSG-PAC1 cells express the pro-acinar markers SOX10 and aquaporin-5. Using the mSG-PAC1 cell line, we demonstrate that FGF2 regulates specific steps during acinar cell maturation. FGF2 up-regulates aquaporin-5 and the expression of the α3 and α6 subunits of the α3β1 and α6β1 integrins that are known to promote SMG morphogenesis and differentiation. mSG-DUC1 and mSG-PAC1 cells were derived from genetically modified mice, homozygous for floxed alleles of the integrin α3 subunit. Similar to SMGs from α3-null mice, deletion of α3 alleles in mSG-PAC1 cells results in the up-regulation of E-cadherin and the down-regulation of CDC42. Our data indicate that mSG-DUC1 and mSG-PAC1 cells will serve as important tools to gain mechanistic insight into salivary gland morphogenesis and differentiation.

## Introduction

Permanent salivary gland damage is a consequence of radiation therapy used for head and neck cancer treatment, which can negatively impact a patient’s quality of life^1^. Such damage impedes saliva production and output by destroying the acinar cells of the gland^1^. This leads to a clinical disorder known as Xerostomia, characterized by impaired digestion and speech, as well as an increased occurrence of dental caries^1^. While a significant clinical problem, there is no known cure. Currently, a major emphasis is on the development of regeneration therapies with a particular emphasis on restoring saliva-producing acinar cells. Although promising advances have been made in this regard, hurdles still remain.

The murine submandibular salivary gland (SMG) is a powerful experimental tool, as it permits the use of mouse genetic models, *ex vivo* SMG cultures, and organoids to identify mechanisms that regulate salivary gland morphogenesis and differentiation^2–6^. This model has been vital in demonstrating that growth factors, released from the mesenchyme, act on the epithelium in a paracrine fashion during morphogenesis and differentiation. In particular, members of the fibroblast growth factor (FGF) family, including FGF2, FGF7, and FGF10 are reported to be integral factors that promote morphogenesis^7^. To tease apart the individual contributions of these factors, epithelial rudiments were separated from the mesenchyme of embryonic SMGs to observe the effects of individual FGF family members^7,8^. The addition of FGF10 enhanced ductal elongation in the epithelial compartment, while stimulation with either FGF2 or FGF7 promoted epithelial budding^9,10^. Notably, the SMG model has also revealed how interactions between integrins and the basement membrane contribute to proper morphogenesis and differentiation of the SMG^11–14^. Integrins are α/β heterodimeric transmembrane receptors that function in both cell adhesion and signal transduction^15^. A subset of integrins binds to laminins, which are α/β/ϒ heterotrimeric proteins that are critical components of the basement membrane^16^. Branching morphogenesis is severely inhibited in glands lacking both α3 and α6 subunits of the α3β1 and α6β1 laminin-binding integrins^11^, whereas differentiation of the gland, particularly the acinar compartment, is defective at E18 in embryos lacking the α3β1 integrin^12^. The α3 and α6 integrins bind to sites present on the α chains of laminin heterotrimers^16^. The addition of function-blocking antibodies to the laminin α1 chain inhibits branching morphogenesis in *ex vivo* culture, whereas the global deletion of the laminin α5 chain inhibits both the morphogenesis and differentiation of the gland^11,13^.

Murine SMGs have also been used to identify progenitor populations in the gland and to test the ability of these cells to repair damaged tissue^17–24^. This model has also been used to develop culture conditions that allow the expansion of populations of cells with stem cell characteristics^25,26^. However, more studies are needed to identify signaling pathways and culture conditions that can promote the differentiation of specific cell types of the salivary glands. The availability of a pro-acinar cell line would provide a novel reagent to identify signaling pathways that promote acinar cell maturation. Although several immortalized cell lines have been established from the salivary gland^27–30^, a pro-acinar cell line has not yet been described.

Our goal in this study was to establish a pro-acinar cell line from the murine SMG to study mechanisms that regulate acinar cell differentiation. We report the establishment and characterization of both a pro-acinar, and a ductal cell line. Our data indicate that the mSG-DUC1 ductal cell line expresses the late stage ductal markers keratin-7 (K7) and keratin-19 (K19) and forms three-dimensional (3-D) structures in a matrix containing basement membrane components. Our mSG-PAC1 cell line expresses the pro-acinar/acinar markers aquaporin-5 (Aqp-5) and SOX10. Treatment of mSG-PAC1 cells with FGF2 leads to morphological changes in 3-D culture and increased expression of E-cadherin, the integrin α3 and α6 subunits, as well as Aqp-5. Since our cell lines were established from transgenic mice carrying floxed alleles of the integrin α3 subunit^31^, we tested the effect of α3 deletion in our pro-acinar cell line. Our data indicate that the lack of α3β1 integrins in mSG-PAC1 cells recapitulates a subset of phenotypes observed in SMGs from α3-null mice^12^.

## Results

### Establishment of ductal and pro-acinar cell lines

**Figure 1:**
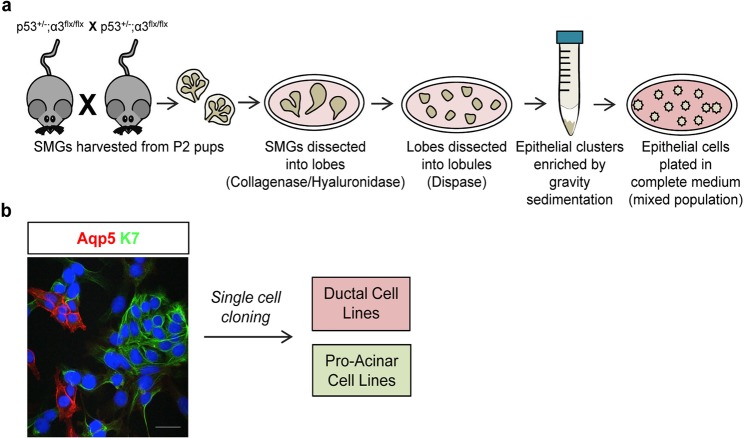
Generation of cell lines. (**a**) A schematic representation of the experimental method used to enrich epithelial cells from dissociated SMGs. **(b)** The original population of epithelial cells was a mixed population of Aqp5 positive and K7 positive cells. Shown is a maximum projection of ten confocal z-slices acquired in 0.4 μm steps with a 40X objective. Size bar, 50 μm.

Although mouse developmental and *ex vivo* studies have provided important insights into the regulation of salivary gland morphogenesis and the identification of progenitor cells, much remains to be learned about the regulation of acinar cell differentiation. The availability of salivary gland epithelial cell lines, particularly a pro-acinar cell line, would provide an important tool for studies aimed at the further understanding of this process. For this purpose, we generated a pro-acinar cell line, and in the process a ductal cell line, from the murine salivary gland. We crossed mice heterozygous for a p53-null allele (Trp53)^32^, and homozygous for the floxed integrin α3 subunit allele (Itga3)^31,33^, using a strategy that was described previously to cross null alleles of the closely linked Trp53 and Itga3 genes^31,33^ (Fig. 1a). SMGs were collected from eight pups at postnatal day 2, and primary cells were isolated from the 16 glands and pooled. Serial passaging led to outgrowth of a p53-null cell population, which was a mixture of cells expressing the ductal marker, K7^34^ and pro-acinar/acinar marker, Aqp5^35^ as assayed by immunofluorescence microscopy (Fig. 1b). Aqp5 is expressed in developing pro-acinar cells and is detectable at the protein level by ICC at embryonic day 15 (E15) when the SMG buds begin to differentiate into pro-acini that do not yet express secretory proteins^36^. Individual clones were isolated and expanded as immortalized cell lines. A pure ductal and a pure pro-acinar cell line were then established by sequential cloning steps. As expected, these clonal lines lacked the wild-type p53 allele (Fig. S1b).

### Characterization of the ductal and pro-acinar cell lines

**Figure 2:**
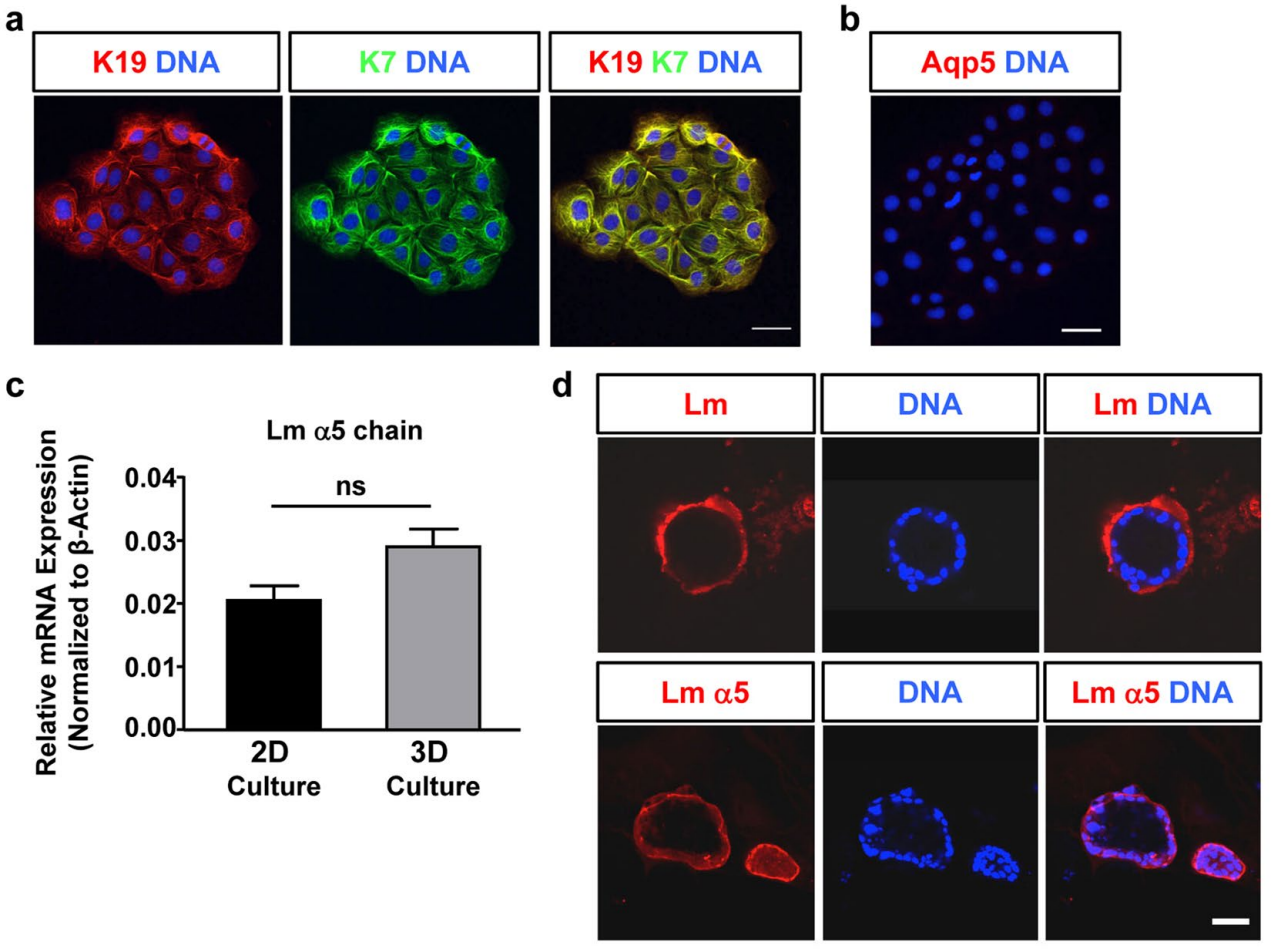
mSG-DUC1 cells express ductal markers & recapitulate ductal morphogenesis in a Matrigel/collagen I matrix. (**a**,**b**) Representative images of mSG-DUC1 cells stained for K7, K19, and Aqp5 acquired at 40X. Images are represented as maximum projection images of ten z-slices taken in 0.4 μm steps. Size bar, 50 μm. (**c**) Laminin (Lm) α5 mRNA expression in mSG-DUC1 cells cultured for five days on tissue culture plastic or in a Matrigel/collagen I matrix for seven days. Data are plotted as the mean ± s.e.m from three independent experiments; ns, not significant. **(d)** Representative images of mSG-DUC1 cells cultured in Matrigel/collagen I 3D culture for seven days and stained with a polyclonal antibody to laminin-111 or a monoclonal antibody to the laminin α5 chain. Images were acquired at 40X and are maximum projections of five z-slices taken in 0.4 μm steps.

To identify pure ductal and pure acinar cell lines, clones were analyzed for the expression of the ductal markers, K7 and K19^34^ and the acinar marker Aqp-5^35^ by immunofluorescence microscopy. We identified one cell line, mSG-DUC1, which co-expresses the ductal keratins (Fig. 2a), but not Aqp-5 (Fig. 2b). Since previous studies demonstrated that ductal epithelial cells form three-dimensional spheroids with hollow lumens in a three-dimensional (3-D) matrix (For example see^37^), we cultured mSG-DUC1 cells in a 3D matrix containing a mixture of Matrigel and collagen I, which was shown previously to promote epithelial morphogenesis^38,39^. First, we examined the expression of the α1 and α5 laminin chains, as the α5 laminin chain is known promote lumen formation^11^. Analysis by qPCR indicated that mSG-DUC1 cells express the α5 chain, whereas the α1 chain is not expressed (Fig. 2c and data not shown). Additionally, mSG-DUC1 cells form spheroids with well-defined lumens when cultured for seven days in the 3-D matrix described above (Fig. 2d). The basal surface of these spheroids stained positively with a monoclonal antibody to the α5 laminin chain, as well as with a polyclonal antibody to the laminin α1, β1 and γ1 chains (Fig. 2d) consistent with the expression of laminin 511. However, it is important to note that because the Matrigel contains laminin 111 and composed a portion of the 3-D matrix used in these studies, the concentration at the basal cell surface may be in part due to the reorganization of the exogenously provided laminin. Thus, mSG-DUC1 cells exhibit many of the properties of ductal epithelial cells.

**Figure 3:**
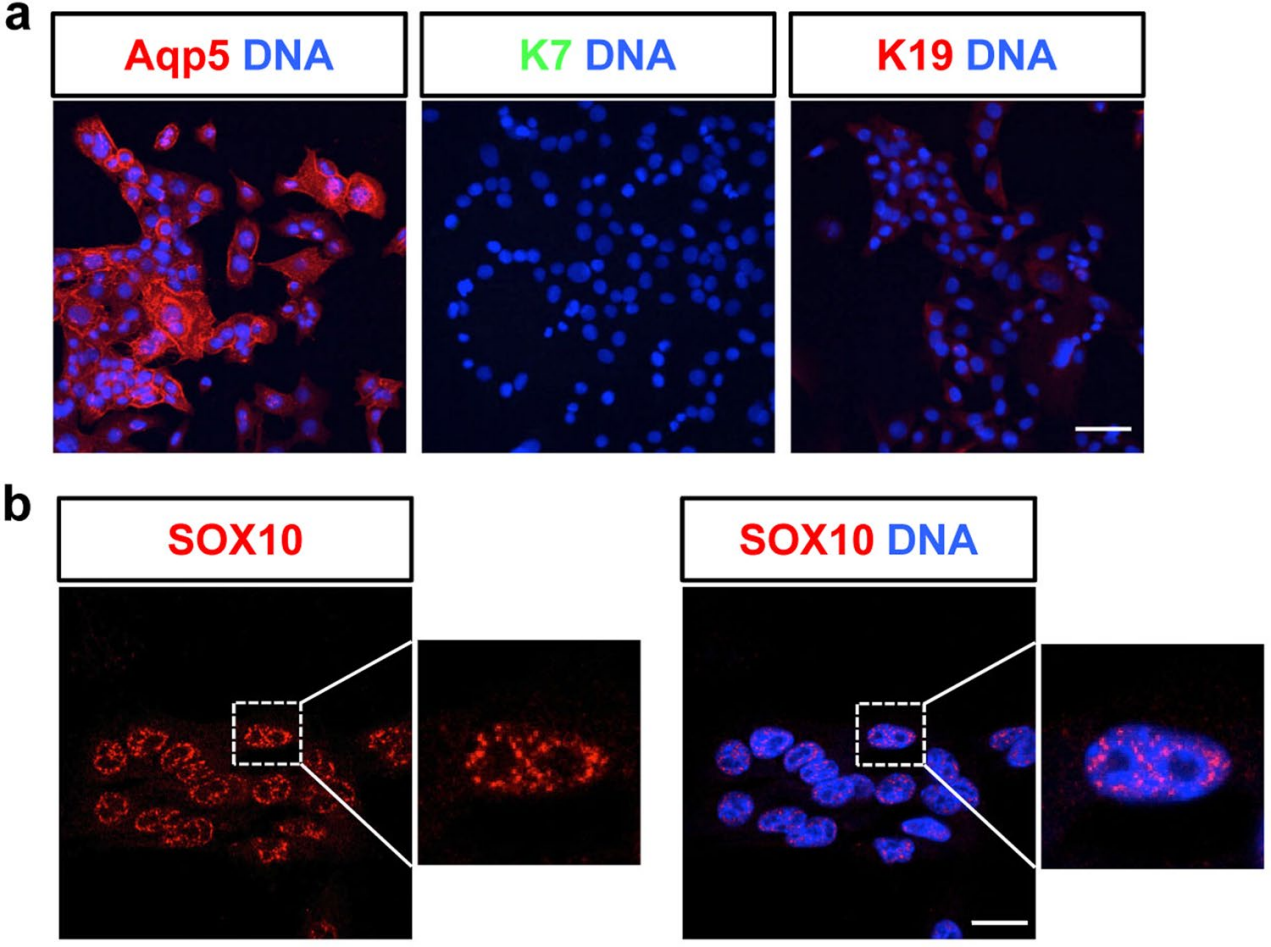
mSG-PAC1 cells express pro-acinar markers. (**a**) Representative images of mSG-PAC1 cells stained for Aqp5, K7, and K19. Images were acquired at 40X and are maximum projections of ten z-slices taken in 0.4 μm z-steps. Size bar, 50 μm. (**b**) Representative image of mSG-PAC1 cells stained for SOX10 is maximum projection of five z-slices acquired at 100X in 0.15 μm steps. Size bar, 20 μm.

An additional cell line that was characterized, mSG-PAC1, expresses the pro-acinar marker Aqp-5 (Fig. 3a). Importantly, mSG-PAC1 cells do not express the ductal markers K7 and K19 (Fig. 3a). mSG-PAC1 cells also express the pro-acinar transcription factor SOX10^4,17^ in the nucleus (Fig. 3b). Notably, mSG-PAC1 cells do not express markers for more mature acinar cells^36,40^, such as Mucin 10 (MUC10) and Mucin 19 (MUC19) (data not shown), consistent with their pro-acinar phenotype. Because acinar cells play a critical role in salivary gland function, we focused our additional studies on mSG-PAC1 cells.

### Fibroblast growth factor 2 (FGF2) promotes the expression of cell-cell and cell-matrix receptors, as well as Aqp5, in mSG-PAC1 cells

**Figure 4:**
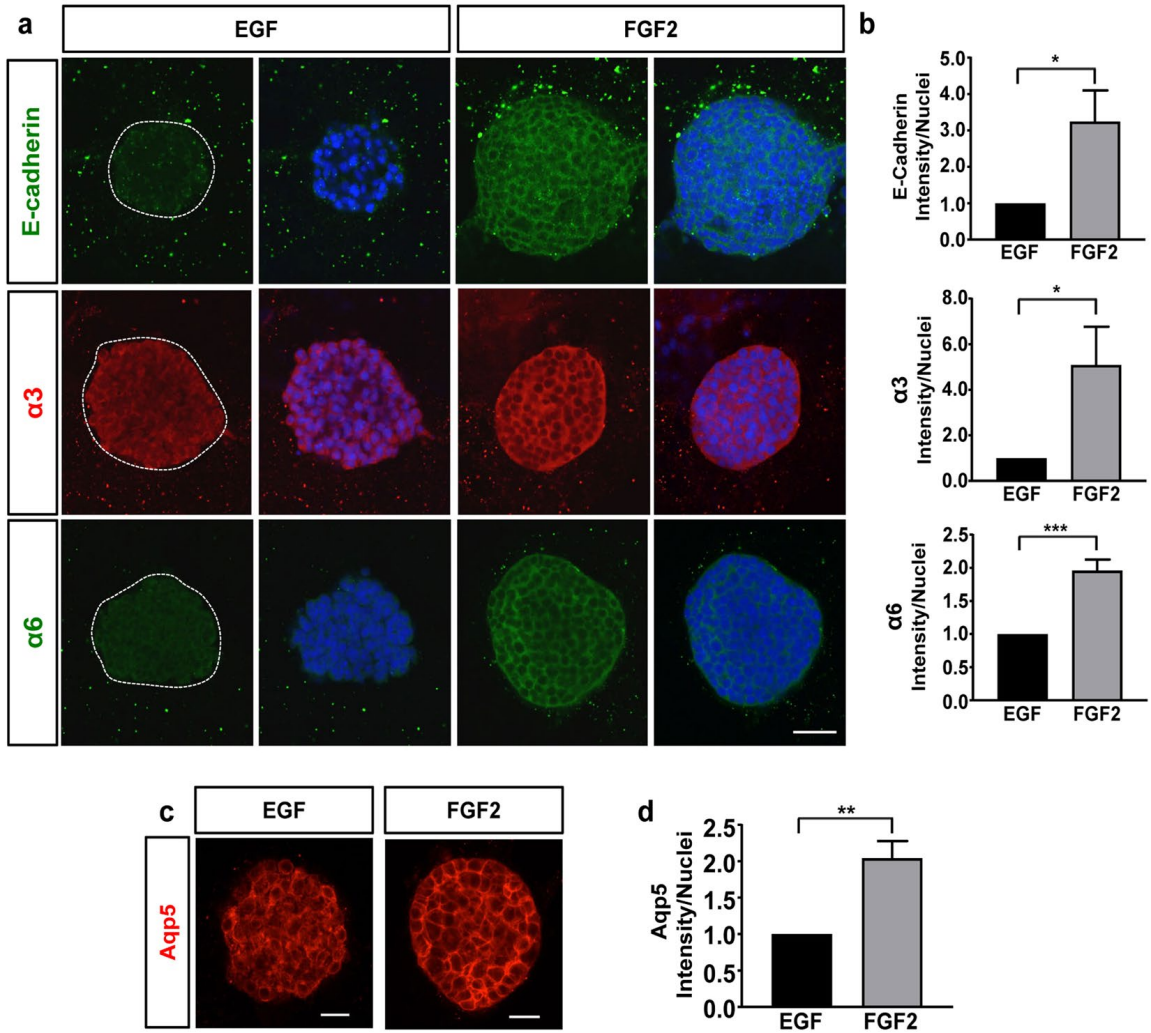
FGF2 promotes the expression of E-cadherin, laminin-binding integrins, & Aqp5. Representative confocal images of mSG-PAC1 spheroids cultured in EGF or FGF2-containing medium for five days in a Matrigel/collagen I matrix and stained for **(a)** E-cadherin, integrin α3 subunit, or integrin α6 subunit and DNA, or **(c)** Aqp5. Images are maximum projection images of five z-slices acquired at 40X taken in 0.4 μm steps. (**b & d**) Fluorescence intensity was normalized to the number of nuclei per field from 13 spheres from three independent experiments and plotted relative to the expression in EGF ± s.e.m. Size bar, 50 μm. Data was analyzed by Student’s T-test. P < 0.05–0.0001, as indicated.

**Figure 5:**
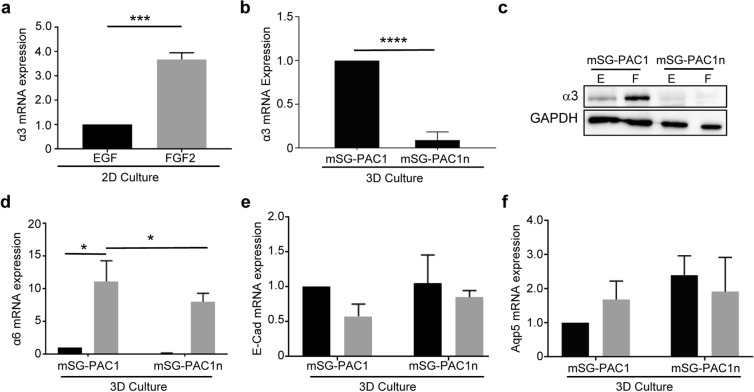
Knockout of α3 integrins in mSG-PAC1 cells inhibits mRNA expression of α6 integrins, but not the expression of E-Cadherin or Aqp5 mRNA in response to FGF2. Cells were incubated in medium containing EGF (black bars) or FGF2 (gray bars) **(a)** Expression of α3 mRNA in mSG-PAC1 cells cultured on tissue culture plastic in the presence of EGF or FGF2 for five days. Expression was normalized to β-actin and then plotted ± s.e.m. as the fold change in FGF2 normalized to expression in EGF. Data are from three independent experiments analyzed by Student’s T-Test. ***p < 0.001. (**b**) Expression of α3 mRNA in mSG-PAC1 and mSG-PAC1n (α3-deleted) cells cultured in a Matrigel/collagen I matrix in the presence of EGF for five days. Expression of α3 was normalized to β-actin and then plotted ± s.e.m. as the fold decrease in mSG-PAC1n compared to mSG-PAC1 cells. Data are from three independent experiments analyzed by Student’s T-test. ***p < 0.001. (**c**) Representative western blot probed for the α3 subunit in mSG-PAC1 and mSG-PAC1n cells cultured on tissue culture plastic for four days. The image of the full-length blot is provided in Supplementary Fig. S6. (**d**) Expression of α6 mRNA in mSG-PAC1 and mSG-PAC1n cells cultured as in panel B in the presence of EGF or FGF2 for five days. Data are from three independent experiments analyzed by two-way ANOVA. p < 0.05. Tukey’s post-hoc analysis was performed to correct for multiple comparisons. (**e**,**f**) Expression of E cadherin mRNA and Aqp5 mRNA in mSG-PAC1 and mSG-PAC1n cells cultured as described in panel D. Data are from three independent experiments analyzed by two-way ANOVA. Differences are not significant.

Previous studies have identified critical roles for E-cadherin, laminins and laminin-binding integrins in SMG morphogenesis employing a variety of experimental approaches, including transgenic mouse models, RNA interference, and function-blocking antibodies^11–13,41^. The global knockout of both α6 and α3 integrins, or the α5 chain of laminin was shown to result in defective branching morphogenesis^11^. Consistent with these studies, application of function-blocking antibodies targeting laminin-111, as well as α6 or β1 integrins was also shown to result in aberrant morphogenesis. Similarly, use of function-blocking antibodies or siRNA targeting E-cadherin was shown to inhibit SMG branching^41,42^. Since these adhesion proteins play important roles during salivary gland development, we examined their expression in mSG-PAC1 cells. Interestingly, when cultured in MCF10A medium, which contains EGF, these cells exhibited low expression of E-cadherin, and α3 and α6 integrins (Fig. 4a). Since previous studies have identified members of the FGF family, including FGF2, as regulators of morphogenesis in the salivary gland^7,10^, and others have cultured primary salivary gland epithelial cells in medium containing FGF2^25^, we tested whether adding FGF2 to our culture medium in place of EGF affected the expression of these adhesion proteins by mSG-PAC1 cells. We analyzed their expression in spheroids by immunofluorence microscopy (Fig. 4a) and quantified the change in fluorescence intensity per nuclei (Fig. 4b). Cells cultured in FGF2 for five days exhibited enhanced expression of E-cadherin, and the α3 and α6 integrin subunits, with E-cadherin increasing by a factor of three, and α3 and α6 increasing by factors of five and two respectively (Fig. 4b). Additionally, the spheroids formed in a 3-D matrix in the presence of FGF2 showed an increase in Aqp5 expression (Fig. 4c) and exhibited a more organized layer of basal columnar cells than in the presence of FGF2 suggestive of a stronger or more organized cell-cell and/or cell matrix adhesions, which is easily visualized in Fig. 4c. Similar to the effects on protein expression, FGF2 also increased the expression of α3 and α6 mRNA transcripts (Fig. 5a,d) Taken together, these results indicate that FGF2 leads to morphological changes in 3-D culture and promotes an increase in the levels of a subset of cell-cell and cell-matrix receptors in mSG-PAC1 cells.

### Integrin α3β1 in pro-acinar cells

**Figure 6:**
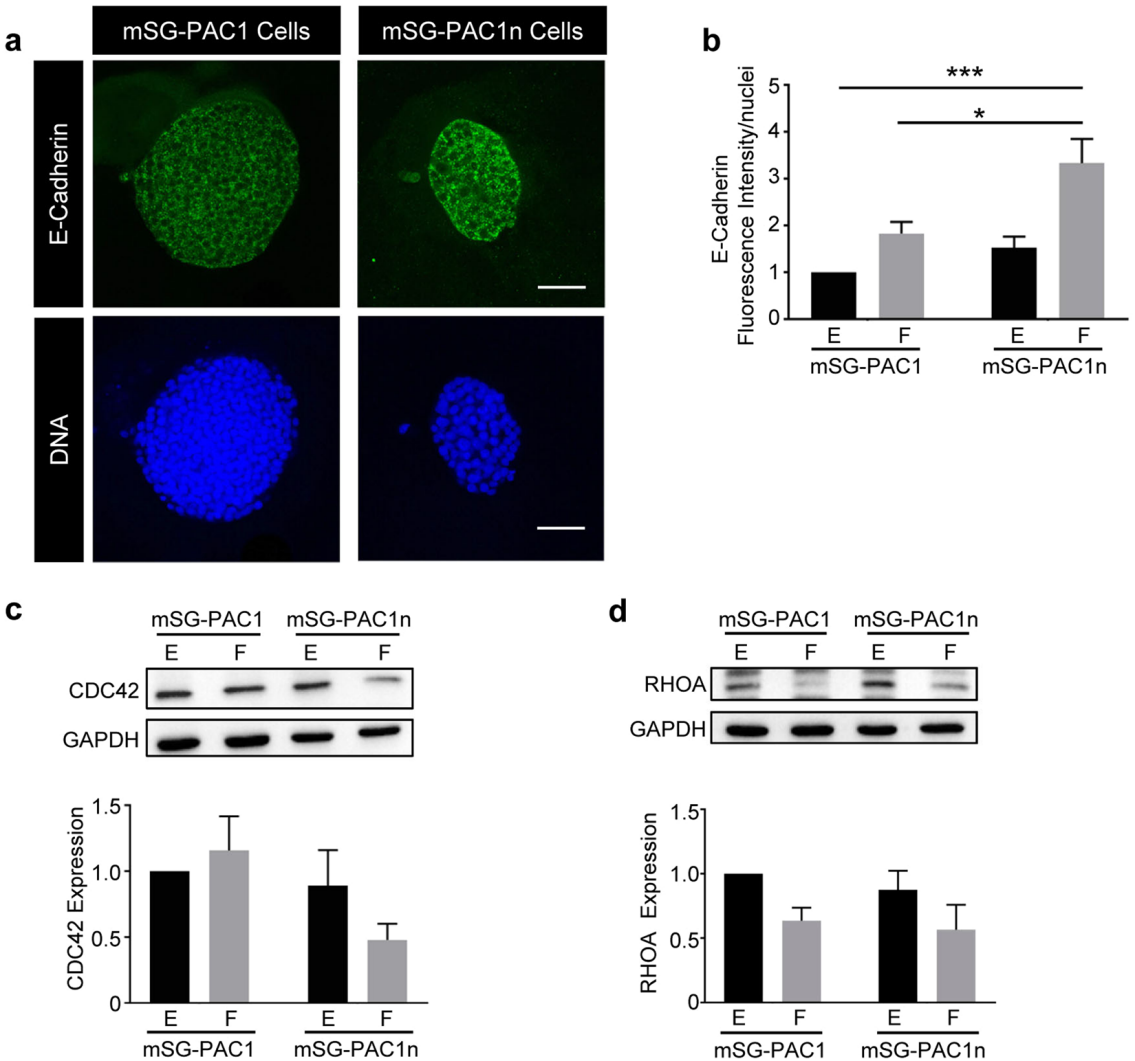
Loss of Integrin α3 in pro-acinar cells results in changes in E-cadherin and CDC42. mSG-PAC1 and mSG-PAC1n cells were cultured in a Matrigel/collagen I matrix for five days in the presence of EGF or FGF2 and stained for E cadherin. **(a)** Representative confocal images of mSG-PAC1and mSG-PAC1n cells in a Matrigel/collagen I matrix cultured in the presence of FGF2 and stained for E cadherin. Images are maximum projections of five z-slices acquired at 40X, in 0.40 um steps. Images of spheres cultured in EGF are not shown as the expression of E-cadherin is extremely low. **(b)** Fluorescence intensity of E-cadherin expression was analyzed for all conditions and normalized to the number of nuclei per field from 15 spheres from three independent experiments. Data is plotted relative to the expression in mSG-PAC1 in EGF ± s.e.m. Data is analyzed by two-way ANOVA. p < 0.05–0.0001, as indicated. Tukey’s post-hoc analysis was performed to correct for multiple comparisons. **(c**,**d)** Representative western blots of **(c)** CDC42 and **(d)** RHOA expression by mSG-PAC1 and mSG-PAC1n cells cultured for four days on tissue culture plastic in the presence of EGF or FGF2. Images of full-length blots are provided in Supplementary Figs 6 and 7, respectively. Relative expression of **(c)** CDC42 and **(d)** RHOA was quantitated from three independent experiments using densitometry analysis and plotted as ± s.e.m as the fold change normalized to expression in mSG-PAC1 in EGF.

The contribution of α3β1 integrins to SMG morphogenesis and differentiation was previously examined using transgenic mice with a global deletion of the integrin α3 subunit^12^. Phenotypes described at E18 included defects in apical-basal polarity, basement membrane assembly, and acinar cell differentiation, as well as changes in the expression of E-cadherin, CDC42, and RHOA. Since our cell lines have floxed integrin α3 subunit alleles (Fig. S1c), we tested whether the deletion of α3 integrins in mSG-PAC1 affects the basal (EGF) or the increase in gene expression in response to FGF2. To delete the integrin α3 subunit alleles, we infected mSG-PAC1 cells with a Cre-recombinase-expressing adenovirus, and cloned infected cells by limiting dilution. We confirmed ablation of α3 expression in the resulting mSG-PAC1n cell line by qPCR (Fig. 5b) and western blotting (Fig. 5c). We then tested whether the lack of α3β1 integrins affects the basal (EGF) or the FGF-induced increase in α6, E-cadherin, or Aqp-5 expression in 3-D culture. The loss of α3β1 inhibited the overall expression of α6 mRNA; however, FGF2 still increased α6 mRNA, although to a much lower overall level (Fig. 5d). The expression of E-cadherin and Aqp-5 mRNA was not affected by the lack of α3 (Fig. 5e,f). Interestingly, the expression of E-cadherin increased significantly in response to FGF in mSG-PAC1n cells (Fig. 6a,b), suggesting that α3β1 negatively regulates E-cadherin protein expression in this context.

Protein levels of the RHO-family GTPases, CDC42 and RHOA were reduced in α3-null SMGs^12^, where the loss of their expression in either the mesenchymal or epithelial compartments could affect morphogenesis. To determine whether α3β1 regulates expression of these two GTPases in our pro-acinar cell line, we performed immunoblots on lysates of the mSG-PAC1 and mSG-PAC1n cell lines. Our results indicate that CDC42 protein levels are consistently reduced in mSG-PAC1n cells (Fig. 6c), although the effect was not as dramatic as observed in α3-null SMGs. RHOA expression was inhibited in response to FGF2, but this change in expression was independent of α3 integrins (Fig. 6d). Thus, the lack α3 integrins in mSG-PAC1 cells recapitulates a subset of phenotypes observed in SMGs from α3-null mice^12^

## Discussion

In this study, we report the isolation and characterization of novel salivary gland epithelial cell lines. Based on the differentiation markers expressed during morphogenesis, mSG-PAC1 cells are similar to pro-acinar cells at the canalicular stage late in embryogenesis^36^. Thus, our mSG-PAC1 cells represent the first reported pro-acinar immortalized cell line. The ductal cell line (mSG-DUC1) expresses late stage ductal differentiation markers, K7 and K19^34^, and has the ability to form three-dimensional structures containing lumens.

Paracrine signaling between the mesenchyme and the epithelium is important for proper development of the salivary gland^7,10^. Several members of the FGF family, including FGF2, are secreted by the mesenchyme to promote epithelial morphogenesis and differentiation^7,8^. Interestingly, the exogenous addition of FGF2 to our pro-acinar cell line increased the expression of the α3 and α6 laminin-binding integrins, as well as E-cadherin and Aqp-5. Notably, FGF2 increased both the mRNA and protein levels of the α3 and α6 integrin subunits; in contrast, E-cadherin and Aqp-5 protein expression increased without a change in mRNA expression, suggesting that FGF2 promotes gene expression in the epithelium of developing gland. It is well recognized that E-cadherin protein levels are regulated by internalization and degradation^43^; however, it is unclear whether FGF2 regulates E-cadherin levels in our cells by these or alternative mechanisms. Interestingly, a previous study showed that Aqp-5 could be regulated post-transcriptionally, with an increase in Aqp-5 translation occurring when cells are cultured in Matrigel independent of FGF2^44^. Recent work has reported that there is significant crosstalk between basement membrane components and the mesenchyme to promote the differentiation of pro-acinar cells of the SMG^6^. Consistent with our work, they report that combination of a laminin-rich matrix supplemented with FGF2 is sufficient to promote the expression of Aqp-5 in E16 epithelial clusters cultured *ex vivo*^6^. However, it is not known whether this regulation of Aqp-5 occurs transcriptionally or post-transcriptionally. The mechanisms that regulate Aqp-5 expression are important topics for future studies.

FGF2 also regulates the morphology of spheroids formed when mSG-PAC1 cells are cultured in a Matrigel/collagen I matrix. In these conditions, spheroids adopt an outer layer of columnar cells, a phenotype that is first observed at E13 during development^41,45^, suggesting that FGF2 may be important to the crosstalk between the mesenchyme and epithelial compartments in some contexts. Since stimulation of mSG-PAC1 cells with FGF2 promotes the expression and surface localization of epithelial adhesion molecules, these phenotypes are likely interconnected. Previous studies have demonstrated crosstalk between other FGFs and integrins during SMG morphogenesis^11^. Our data implicate FGF2 as an additional growth factor involved in this process. However, the mechanism of FGF2 effects may be context dependent, as our previous studies indicated that FGF2 has an autocrine effect on the mesenchyme, and thus has a more indirect effect on promoting Aqp-5 expression in E16 epithelium^6^.

Our cell lines were isolated from transgenic mice homozygous for the floxed integrin α3 subunit allele^31^. To date, the role of α3β1 integrins in SMG development has only been studied in the context of global α3- null mice or mice null for both α3 and α6 alleles^11,12^, which precluded the determination whether α3β1 integrins play specific roles in individual cell types present in the SMG. The identification of cell-type specific contributions is important, as crosstalk between the mesenchyme and epithelial compartments is critical to SMG differentiation and genetic models with global α3 deletion cannot account for such crosstalk. Deletion of the floxed α3 alleles in our pro-acinar cells resulted in several phenotypes that were previously reported for α3- null glands, including an increase in E-cadherin expression and a decrease in CDC42 expression^12^, suggesting that these phenotypes are due, at least in part to cell autonomous effects of the loss of α3β1 integrins.

The loss of E-cadherin expression or function leads to the inhibition of branching morphogenesis and impaired ductal development; however, the consequences of increased cadherin expression to the morphogenesis or differentiation of the SMG is not easily appreciated. The effect of decreased expression of CDC42 is more easily appreciated. CDC42 regulates apical basal polarity and is required for the establishment of epithelial polarity during early development^46,47^. Additionally, the expression of CDC42 has been shown to regulate the stability of the epithelial basement membrane in the skin^48^, which is also regulated by the α3β1 integrin^49,50^. Thus, the down-regulation of CDC42 in α3-null glands helps explain defects in basement membrane assembly observed α3-null SMGs^12^; however, it is unclear whether the changes in CDC42 protein expression that we observed in mSG-PAC1n are sufficient to alter these processes.

Deletion of α3 integrins was also shown to down-regulate the expression of RHOA^12^. In the developing salivary gland, as well as in the lung and kidney, signaling downstream of RHOA regulates branching morphogenesis^51–53^. Interestingly, FGF2 is sufficient to suppress RHOA expression in mSG-PAC1 cells and this regulation is independent of α3 integrins. This is surprising, as the inhibition of RHO kinase disrupts the polarized deposition of basement membrane, as well as the columnar morphology of the outer layer of epithelial cells^54^. Thus in mSG-PAC1 cells, FGF2 signaling may somehow bypass the requirement for RHOA signaling in the maintenance of this columnar morphology and regulation may be different in P2-derived cells or in the absence of p53.

Despite being isolated from P2 glands, mSG-PAC1 cells only express pro-acinar markers. This could be due to the loss of p53. Although the loss of p53 facilitates the establishment of immortalized cell lines, the down-regulation of its activity is also important during development. For example, the inactivation of p53 is imperative to proper branching morphogenesis in the developing kidney^55^, and the down-regulation of p53 is important for regenerative processes^56^ and for maintenance of stemness^57^. The loss of p53 expression in our cells might have resulted in a pro-acinar phenotype by either the de-differentiation of P2 acinar cells, or the immortalization of an acinar progenitor cell population. In either case, the plasticity exhibited by these cells will allow them to be manipulated in culture to recapitulate some of the developmental and differentiation aspects of the SMG. A G-banded Karyotype has been performed on mSG-PAC1 cells, and has been included in Fig. S2.

In summary, we have described the isolation and characterization of the first pure pro-acinar cell line derived from the murine submandibular salivary gland. These cells will be a valuable tool in future studies to dissect the molecular mechanisms that regulate acinar differentiation and branching morphogenesis through the addition of specific factors alone or in combination with mesenchyme.

## Material and Methods

### Isolation of primary SMG and establishment of immortalized epithelial cell lines

Since the Trp53 and Itga3 are closely linked on mouse chromosome 11, we first generated mice that carry the p53-null allele^32^ and the floxed integrin α3 subunit allele^31,33^ on the same chromosome copy in a mixed background, using a breeding strategy that we described previously to similarly link the p53-null and α3-null mutations^31,33^. We next crossed mice heterozygous for the p53-null allele and homozygous for the floxed integrin α3 subunit allele, then harvested a total of 16 submandibular salivary glands (SMGs) from 8 pups at postnatal day 2, according to protocols approved by the Institutional Animal Care and Use Committees (IACUC) of Albany Medical College. SMGs were collected from eight pups at postnatal day 2. Although only 3 of the 8 neonates were homozygous for the p53-null mutation (Fig. S1a), we isolated epithelial cells from SMGs from all 8 pups and pooled them in culture to maintain high cell densities through early serial passaging. Glands were enzymatically dissected into lobes following a 20 min incubation at 37**°**C in DMEM containing 600 U/ml collagenase and 200 U/ml hyaluronidase (Stem Cell Technologies, #7912), and then further dissected into lobules following a 30 min incubation at 37**°**C in 0.8 U/ml Dispase II (Life Technologies, #17105041). Dissected lobules were triturated to dissociate epithelial clusters. Gravity sedimentation was repeated several times to enrich for the epithelial cells. The resulting cells were cultured in a tissue culture incubator maintained at 37**°**C with 5% CO_2_ in a modification of the culture medium previously described for the isolation the mammary epithelial cell line MCF10A^37,58^ and consists of DMEM/F12 supplemented with 5% donor horse serum (Atlanta Biologicals, #S12150), 100 U/ml penicillin/streptomycin (Hyclone, #SV30010), 20 ng/ml human recombinant EGF (Gibco, #PHG0311L), 100 ng/ml Cholera Toxin (Sigma, #C8052), 2.5 μg/ml hydrocortisone (Sigma, #H0396), and 20 μg/ml human insulin (Sigma, #I9278). Cells were cultured at a high density through 11 serial passages. To isolate pure populations of ductal and pro-acinar cells, epithelial cells were plated at low density and twenty-six single colonies were harvested using cloning rings. Pure ductal and pro-acinar populations were then isolated through cloning by limiting dilution.

### Epithelial cell cultures

Once established, cell lines were maintained using culture conditions described above. For cultures supplemented with FGF2, human recombinant EGF was replaced with 100 ng/ml bFGF/FGF2 (Peprotech, #450-33). For three-dimensional (3D) cultures, matrices were prepared in 8-well chamber slides (Corning, #08-774-208). Matrices consisted of a mixture of 60% Matrigel (Corning, #354230, protein concentration ~10 mg/ml, endotoxin <1.5 mg/ml) and 40% collagen (Pure Col Collagen I (3 mg/ml); Advanced BioMatrix, #5005-B, Lot #6260). Approximately, 1000 cells were plated per well and cultured for 5 or 7 days in medium supplemented with 2% Matrigel in the presence of EGF or FGF2, as indicated in the Figure Legends.

### Immunostaining

Cells cultured on glass coverslips were fixed with either 4% paraformaldehyde for 15 min to visualize Aqp-5, the α3 or α6 integrins, E-cadherin, and SOX10 or cold methanol for 5 min to visualize K7, and K19. Cells were permeabilized for 15 min in 0.5% Triton-X-100/PBS and washed in 1X PBS prior to blocking in 2% BSA/PBS for 1 hr. Spheroids in 3D culture were fixed for 20 min in 4% paraformaldehyde, washed in 0.5% PBST, permeabilized in 0.4% Triton-X-100/PBS for 20 min and then washed in 0.5% PBST before blocking in 20% donkey serum. Antibodies were prepared in 3% BSA/PBST. All antibodies and dilutions used for immunofluorescence are listed in Table 1. DRAQ5 (Cell Signaling) was used at a dilution of 1:1000. Coverslips and slides were mounted using SlowFade**®**Gold antifade mounting medium (Life Technologies, #P36930).

**Table 1:** Antibodies.

Antibody	Company	Catalog #	Dilution	Application
**Primary Antibodies**				
AQP5	Alomone Labs	AQP-005	1:200	ICC
Laminin	Abcam	Ab11575	1:400	ICC
Laminin α5	*Gift from Lydia Sorokin*		1:400	ICC
Cytokeratin 7	Abcam	Ab9021	1:100	ICC
Cytokeratin 19, TROMA-III	DSHB	AB-2133570	1:100	ICC
E-cadherin	BD Biosciences	610182	1:200	ICC
Integrin α6	BD Biosciences	555734	1:200	ICC
Integrin α3	*C*.*M*. *DiPersio*		1:200, 1:1000	ICC, WB
Sox10	Santa Cruz	sc-17342	1:200	ICC
Cdc42	Santa Cruz	sc-8401	1:500	WB
RhoA	Santa Cruz	sc-179	1:500	WB
GAPDH	Invitrogen	MA5-15738	1:3000	WB
**Secondary Antibodies**				
AF568 Donkey anti-Rabbit	Alexa-Fluor	A10042	1:1000 (2D) 1:500 (3D)	ICC
AF488 Donkey anti-Mouse	Alexa-Fluor	A21202	1:1000 (2D) 1:500 (3D)	ICC
AF488 Goat anti-Rat	Alexa-Fluor	A11006	1:1000 (2D) 1:500 (3D)	ICC
AF488 Donkey anti-Goat	Alexa-Fluor	A11055	1:1000 (2D) 1:500 (3D)	ICC
AF568 Goat anti-Rat	Alexa-Fluor	A11077	1:1000 (2D) 1:500 (3D)	ICC
AF568 Donkey anti-Goat	Alex-Fluor	A11057	1:1000 (2D) 1:500 (3D)	ICC
DRAQ5	Cell Signaling Technology	4084	1:1000 (2D) 1:500 (3D)	ICC

Immunocytochemistry (ICC).

Western Blotting (WB).

### Genotyping

To obtain DNA for genotyping, tissue or cells were incubated overnight at 55**°**C in 0.1 M Tris-HCl, pH 8.5, 5 mM EDTA, 0.2% SDS, 0.2 mM NaCl and 100 μg/ml proteinase K. Proteinase K was then denatured at 100**°**C for 5 min. Genotyping was performed using the REDTaq® PCR ReadyMix (Sigma, #R2523) and the Biorad iCycler Thermocycler. PCR conditions for the analysis of the p53 and α3^fl/fl^ alleles are provided in Table 2 and PCR primers in Table 3. PCR products were verified on a 2% agarose gel compared to PCR Markers (Promega).

**Table 2:** p53/α3^fl/fl^ PCR Protocol.

Temperature (°C)	Time	# Cycles
94	2 min	1
94	30 sec	33
55	30 sec	
72	45 sec	
72	5 min	1

**Table 3:** PCR Primers.

Allele	Strand	Sequence (5′-3′)
p53 WT allele	Forward	ATGGGAGGCTGCCAGTCCTAACCC
	Reverse	GTGTTTCATTAGTTCCCCACCTTGAC
p53 Null allele	Forward	TTTACGGAGCCCTGGCGCTCGATGT
	Reverse	GTGGGAGGGACAAAAGTTCGAGGCC
Integrin α3 fl/fl allele	Forward	TGATGACTATACCAACCGGAC
	Reverse	ACTCCAAGCCACATATCCTC

### Microscopy

Images were acquired using an inverted Nikon TE2000-E microscope with phase contrast and epifluorescence, a Prior ProScanII motorized stage, and Nikon C1 confocal system with EZC1 and NIS-Elements acquisition software. Confocal images were acquired at 40X or 100X, and are represented as maximum projection images of confocal slices, as indicated in the Figure Legends, taken at 0.4 μm or 0.15 μm, respectively. Fluorescence intensities were quantified using maximum projection images and normalized to total number of nuclei per field using ImageJ Fiji^59^.

### RNA isolation and quantitative PCR (qPCR)

RNA was extracted with TRIzol (Ambion, #15596026) and genomic DNA was removed with TURBO DNaseI (ThermoFisher Scientific, #AM1907) according to the manufacturers’ protocols. cDNA was synthesized from 1 μg of RNA using the iScript Reverse Transcription Supermix kit (Biorad, #1708840). Equal concentrations of cDNA were used in qPCR reactions with iQ SYBR Green Supermix (Biorad, #170-8880). Reactions were run in triplicate using the BioRad CFX96 Real-time system C1000 Touch Thermal Cycler. Ct values were normalized to β-Actin. A list of primer sequences can be found in Table 4.

**Table 4:** qPCR primers.

Gene Product	Strand	Sequence (5′-3′)
Integrin α6	Forward	TGCAGAGGGCGAACAGAAC
	Reverse	GCACACGTCACCACTTTGC
Integrin α3	Forward	CCTCTTCGGCTACTCGGTC
	Reverse	CCGGTTGGTATAGTCATCACCC
AQP5	Forward	AGAAGGAGGTGTGTTCAGTTGC
	Reverse	GCCAGAGTAATGGCCGGAT
E-cadherin	Forward	GACTGGAGTGCCACCACCAAAGAC
	Reverse	CGCCTGTGTACCCTCACCATCGG
Laminin α1	Forward	ATTTAGCCAATGGAAAGTGG
	Reverse	TTTTCTTACAAAGACACGGC
Laminin α5	Forward	TGTTTTTGTACAGCGACTTC
	Reverse	CTACGCTTACATTGACACTC
β-Actin	Forward	GGCTGTATTCCCCTCCATCG
	Reverse	CCAGTTGGTAACAATGCCATGT

### Immunoblotting

Western blot analysis was used to assay the expression of integrin α3 s, RHOA, and CDC42 in mSG-PAC1 and mSG-PAC1n cells (described below) upon stimulation with FGF2. Cells were lysed in mRIPA buffer containing a protease/phosphatase inhibitor cocktail used at a 1:100 dilution. Equal volumes of protein were separated by 10% SDS-PAGE (integrin α3 subunit) or 12% SDS-PAGE (RHOA, CDC42), and transferred onto either 0.4 μm (integrin α3 subunit) or 0.2 μm (RHOA, CDC42) nitrocellulose membrane for analysis. Membranes were blocked in 2% BSA/PBST for 1 hour and then incubated with primary antibody application overnight at 4 °C. Membranes were washed in PBST prior to secondary antibody application. Membranes were exposed to SuperSignal West Pico PLUS Chemiluminescent substrate (Thermo Scientific, #34580) and SuperSignal West Femto Maximum Sensitivity substrate (Thermo Scientific, #34095) prior to imaging on the Biorad ChemiDoc^TM^ MP Imaging System and analysis with Image Lab software. Protein signals were normalized to the expression of GAPDH. All antibodies and dilutions used for immunoblotting are listed in Table 1.

### Deletion of the integrin α3 subunit from the mSG-PAC1 pro-acinar cell line

The α3 floxed alleles were deleted from our pro-acinar cells using an adenovirus for the expression of Cre recombinase. This adenovirus was a gift from Dr. Mingfu Wu (Albany Medical College), and was amplified and purified as previously described^60^. Transduction efficiency was monitored by immunofluorescence using an antibody to Cre Recombinase (Millipore, #MAB3120, Clone 2D8). To obtain maximal deletion of both integrin α3 alleles, multiple adenoviral infections were required. Infected cells were cloned using limiting dilution to generate the mSG-PAC1n α3 knockout cell line. Ablation of integrin α3 expression was confirmed at the mRNA transcript (qPCR) and protein levels (western blot).

### G-Banded karyotype

The G-Banded Karyotype of mSC-PAC1 cells was performed by Cell Line Genetics, Madison, Wisconsin, USA.

### Statistical analysis

Statistical analyses were performed using the GraphPad Prism software employing Student’s t-tests and two-way ANOVAs as indicated in the Figure Legends. Tukey’s post hoc analyses were performed on all ANOVAs to correct for multiple comparisons. P values of <0.05 were deemed statistically significant.

## Supplementary information

Supplemental Information

## References

[1] Vissink A (2010). Clinical management of salivary gland hypofunction and xerostomia in head-and-neck cancer patients: successes and barriers. Int J Radiat Oncol Biol Phys.

[2] Arany S, Catalan MA, Roztocil E, Ovitt CE (2011). Ascl3 knockout and cell ablation models reveal complexity of salivary gland maintenance and regeneration. Dev Biol.

[3] Maruyama EO (2016). Cell-Specific Cre Strains For Genetic Manipulation in Salivary Glands. PLoS One.

[4] Emmerson, E. *et al*. SOX2 regulates acinar cell development in the salivary gland. *Elife* **6**, 10.7554/eLife.26620 (2017). 10.7554/eLife.26620 PMC5498133 28623666

[5] Daley WP (2017). Btbd7 is essential for region-specific epithelial cell dynamics and branching morphogenesis *in vivo*. Development.

[6] Hosseini Zeinab F., Nelson Deirdre A., Moskwa Nicholas, Sfakis Lauren M., Castracane James, Larsen Melinda (2018). FGF2-dependent mesenchyme and laminin-111 are niche factors in salivary gland organoids. Journal of Cell Science.

[7] Hoffman MP (2002). Gene expression profiles of mouse submandibular gland development: FGFR1 regulates branching morphogenesis *in vitro* through BMP- and FGF-dependent mechanisms. Development.

[8] Morita K, Nogawa H (1999). EGF-dependent lobule formation and FGF7-dependent stalk elongation in branching morphogenesis of mouse salivary epithelium *in vitro*. Dev Dyn.

[9] Patel VN (2008). Specific heparan sulfate structures modulate FGF10-mediated submandibular gland epithelial morphogenesis and differentiation. J Biol Chem.

[10] Steinberg Z (2005). FGFR2b signaling regulates *ex vivo* submandibular gland epithelial cell proliferation and branching morphogenesis. Development.

[11] Rebustini IT (2007). Laminin alpha5 is necessary for submandibular gland epithelial morphogenesis and influences FGFR expression through beta1 integrin signaling. Dev Biol.

[12] Menko AS, Kreidberg JA, Ryan TT, Van Bockstaele E, Kukuruzinska MA (2001). Loss of alpha3beta1 integrin function results in an altered differentiation program in the mouse submandibular gland. Dev Dyn.

[13] Kadoya Y (1995). Antibodies against domain E3 of laminin-1 and integrin alpha 6 subunit perturb branching epithelial morphogenesis of submandibular gland, but by different modes. J Cell Biol.

[14] Mathew SS (2014). Integrins promote cytokinesis through the RSK signaling axis. J Cell Sci.

[15] Hynes RO (2002). Integrins: bidirectional, allosteric signaling machines. Cell.

[16] Yurchenco P. D. (2010). Basement Membranes: Cell Scaffoldings and Signaling Platforms. Cold Spring Harbor Perspectives in Biology.

[17] Lombaert IM (2013). Combined KIT and FGFR2b signaling regulates epithelial progenitor expansion during organogenesis. Stem Cell Reports.

[18] Chatzeli L, Gaete M, Tucker AS (2017). Fgf10 and Sox9 are essential for the establishment of distal progenitor cells during mouse salivary gland development. Development.

[19] Chibly AM, Querin L, Harris Z, Limesand KH (2014). Label-retaining cells in the adult murine salivary glands possess characteristics of adult progenitor cells. PLoS One.

[20] Emmerson Elaine, May Alison J, Berthoin Lionel, Cruz‐Pacheco Noel, Nathan Sara, Mattingly Aaron J, Chang Jolie L, Ryan William R, Tward Aaron D, Knox Sarah M (2018). Salivary glands regenerate after radiation injury through SOX2‐mediated secretory cell replacement. EMBO Molecular Medicine.

[21] May Alison J., Cruz-Pacheco Noel, Emmerson Elaine, Gaylord Eliza A., Seidel Kerstin, Nathan Sara, Muench Marcus O., Klein Ophir D., Knox Sarah M. (2018). Diverse progenitor cells preserve salivary gland ductal architecture after radiation-induced damage. Development.

[22] Weng PL, Aure MH, Maruyama T, Ovitt CE (2018). Limited Regeneration of Adult Salivary Glands after Severe Injury Involves Cellular Plasticity. Cell Rep.

[23] Lombaert IM (2008). Rescue of salivary gland function after stem cell transplantation in irradiated glands. PLoS One.

[24] Nanduri LS (2011). Regeneration of irradiated salivary glands with stem cell marker expressing cells. Radiother Oncol.

[25] Maimets M (2016). Long-Term *In Vitro* Expansion of Salivary Gland Stem Cells Driven by Wnt Signals. Stem Cell Reports.

[26] Nanduri LS (2014). Purification and *ex vivo* expansion of fully functional salivary gland stem cells. Stem Cell Reports.

[27] Laoide BM (1996). Immortalised mouse submandibular epithelial cell lines retain polarised structural and functional properties. J Cell Sci.

[28] Quissell DO (1997). Development and characterization of SV40 immortalized rat submandibular acinar cell lines. In Vitro Cell Dev Biol Anim.

[29] Min S (2018). Functional characterization and genomic studies of a novel murine submandibular gland epithelial cell line. PLoS One.

[30] Ikeura K, Kawakita T, Tsunoda K, Nakagawa T, Tsubota K (2016). Characterization of Long-Term Cultured Murine Submandibular Gland Epithelial Cells. PLoS One.

[31] Mitchell K (2009). Alpha3beta1 integrin in epidermis promotes wound angiogenesis and keratinocyte-to-endothelial-cell crosstalk through the induction of MRP3. J Cell Sci.

[32] Donehower LA (1992). Mice deficient for p53 are developmentally normal but susceptible to spontaneous tumours. Nature.

[33] Lamar JM, Pumiglia KM, DiPersio CM (2008). An immortalization-dependent switch in integrin function up-regulates MMP-9 to enhance tumor cell invasion. Cancer Res.

[34] Azevedo RS, de Almeida OP, Kowalski LP, Pires FR (2008). Comparative cytokeratin expression in the different cell types of salivary gland mucoepidermoid carcinoma. Head Neck Pathol.

[35] Larsen HS (2011). Localization of AQP5 during development of the mouse submandibular salivary gland. J Mol Histol.

[36] Nelson DA (2013). Quantitative single cell analysis of cell population dynamics during submandibular salivary gland development and differentiation. Biol Open.

[37] Debnath J, Muthuswamy SK, Brugge JS (2003). Morphogenesis and oncogenesis of MCF-10A mammary epithelial acini grown in three-dimensional basement membrane cultures. Methods.

[38] Kleinman HK (1986). Basement membrane complexes with biological activity. Biochemistry.

[39] Nguyen-Ngoc KV, Ewald AJ (2013). Mammary ductal elongation and myoepithelial migration are regulated by the composition of the extracellular matrix. J Microsc.

[40] Das B, Cash MN, Hand AR, Shivazad A, Culp DJ (2009). Expression of Muc19/Smgc gene products during murine sublingual gland development: cytodifferentiation and maturation of salivary mucous cells. J Histochem Cytochem.

[41] Walker JL (2008). Diverse roles of E-cadherin in the morphogenesis of the submandibular gland: insights into the formation of acinar and ductal structures. Dev Dyn.

[42] Wei C, Larsen M, Hoffman MP, Yamada KM (2007). Self-organization and branching morphogenesis of primary salivary epithelial cells. Tissue Eng.

[43] Bryant DM, Stow JL (2004). The ins and outs of E-cadherin trafficking. Trends Cell Biol.

[44] Maria OM, Maria O, Liu Y, Komarova SV, Tran SD (2011). Matrigel improves functional properties of human submandibular salivary gland cell line. Int J Biochem Cell Biol.

[45] Kadoya Y, Yamashina S (2010). Cellular dynamics of epithelial clefting during branching morphogenesis of the mouse submandibular gland. Dev Dyn.

[46] Etienne-Manneville S (2004). Cdc42–the centre of polarity. J Cell Sci.

[47] Wu X (2007). Cdc42 is crucial for the establishment of epithelial polarity during early mammalian development. Dev Dyn.

[48] Wu X, Quondamatteo F, Brakebusch C (2006). Cdc42 expression in keratinocytes is required for the maintenance of the basement membrane in skin. Matrix Biol.

[49] DiPersio CM, Hodivala-Dilke KM, Jaenisch R, Kreidberg JA, Hynes R (1997). O. alpha3beta1 Integrin is required for normal development of the epidermal basement membrane. J Cell Biol.

[50] Longmate WM (2017). Suppression of integrin alpha3beta1 by alpha9beta1 in the epidermis controls the paracrine resolution of wound angiogenesis. J Cell Biol.

[51] Daley WP, Gulfo KM, Sequeira SJ, Larsen M (2009). Identification of a mechanochemical checkpoint and negative feedback loop regulating branching morphogenesis. Dev Biol.

[52] Moore KA (2005). Control of basement membrane remodeling and epithelial branching morphogenesis in embryonic lung by Rho and cytoskeletal tension. Dev Dyn.

[53] Meyer TN (2006). Rho kinase acts at separate steps in ureteric bud and metanephric mesenchyme morphogenesis during kidney development. Differentiation.

[54] Daley WP (2012). ROCK1-directed basement membrane positioning coordinates epithelial tissue polarity. Development.

[55] Hilliard S, Aboudehen K, Yao X, El-Dahr SS (2011). Tight regulation of p53 activity by Mdm2 is required for ureteric bud growth and branching. Dev Biol.

[56] Yun MH, Gates PB, Brockes JP (2013). Regulation of p53 is critical for vertebrate limb regeneration. Proc Natl Acad Sci USA.

[57] Liu Y (2009). p53 regulates hematopoietic stem cell quiescence. Cell Stem Cell.

[58] Soule HD (1990). Isolation and characterization of a spontaneously immortalized human breast epithelial cell line, MCF-10. Cancer Res.

[59] Schindelin J (2012). Fiji: an open-source platform for biological-image analysis. Nat Methods.

[60] Bartholomew PJ, Jones CW, Benware A, Chernoff J, LaFlamme SE (2005). Regulation of the catalytic activity of PTP1B: roles for cell adhesion, tyrosine residue 66, and proline residues 309 and 310. Exp Cell Res.

