# Supplementary material for: Establishment of a Murine Pro-acinar Cell Line to Characterize Roles for FGF2 and α3β1 Integrins in Regulating Pro-acinar Characteristics
Source: Sci Rep. 2019 Jul 29;9:10984. doi: 10.1038/s41598-019-47387-y (PMC6662831; doi:10.1038/s41598-019-47387-y)
Supplement: Supplementary file 1 — Supplemental Information [file 41598_2019_47387_MOESM1_ESM.pdf]

Supplementary Information

Establishment of a Murine Salivary Pro-Acinar Cell Line to Characterize Roles for FGF2 and α3β1 Integrin in Regulating Pro-Acinar Characteristics

Renée F. Thiemann[1], Deirdre A. Nelson[2], C. Michael DiPersio[3],
Melinda Larsen[2] and Susan E. LaFlamme[1*]

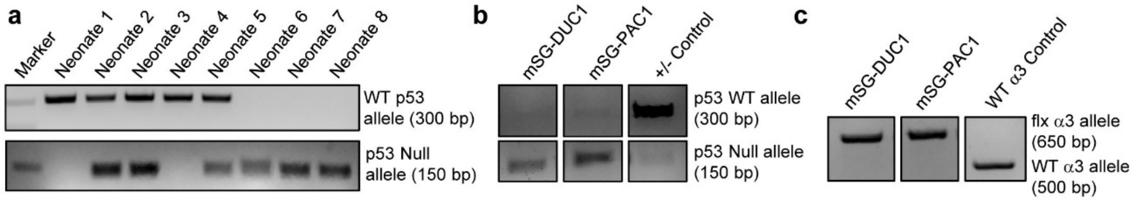

Figure S1. Ductal & pro-acinar cell lines are homozygous for the floxed (flx) integrin α3 subunit allele and p53 null allele. (A) PCR analysis of genomic DNA from P2 neonates used for SMG isolation and dissection. (B) PCR analysis of genomic DNA from the ductal and pro-acinar cell lines for **(B)** the p53 null allele, and **(C)** the integrin α3 floxed allele.

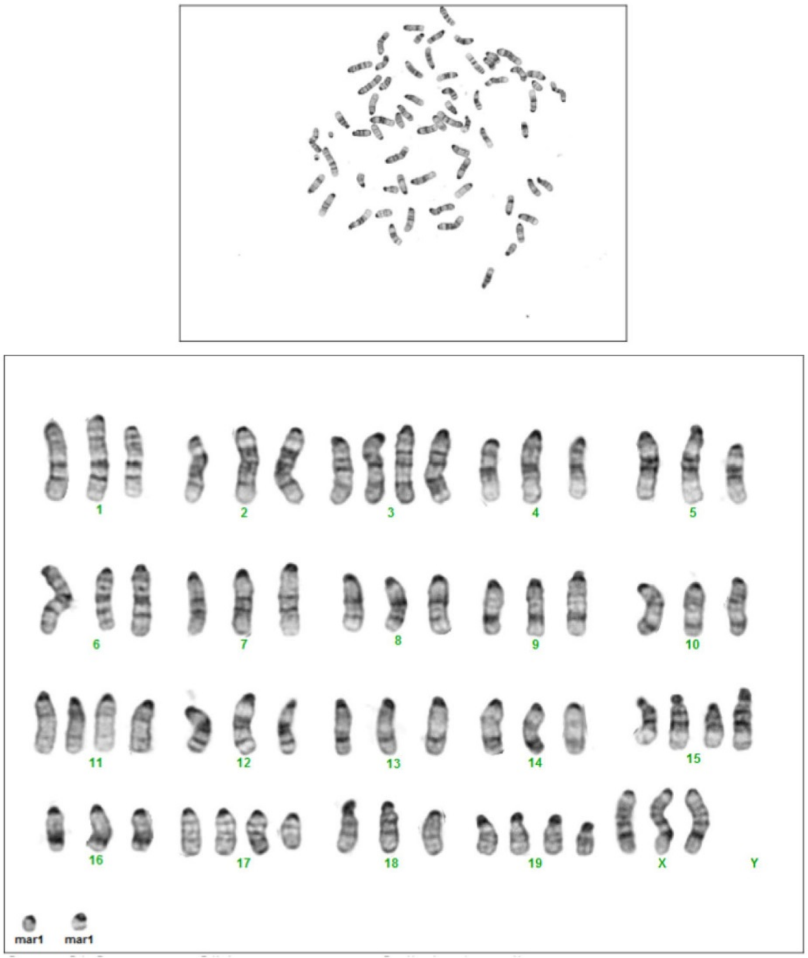

Figure S2. G-banded karyotype of mSG-PAC1 cell line.

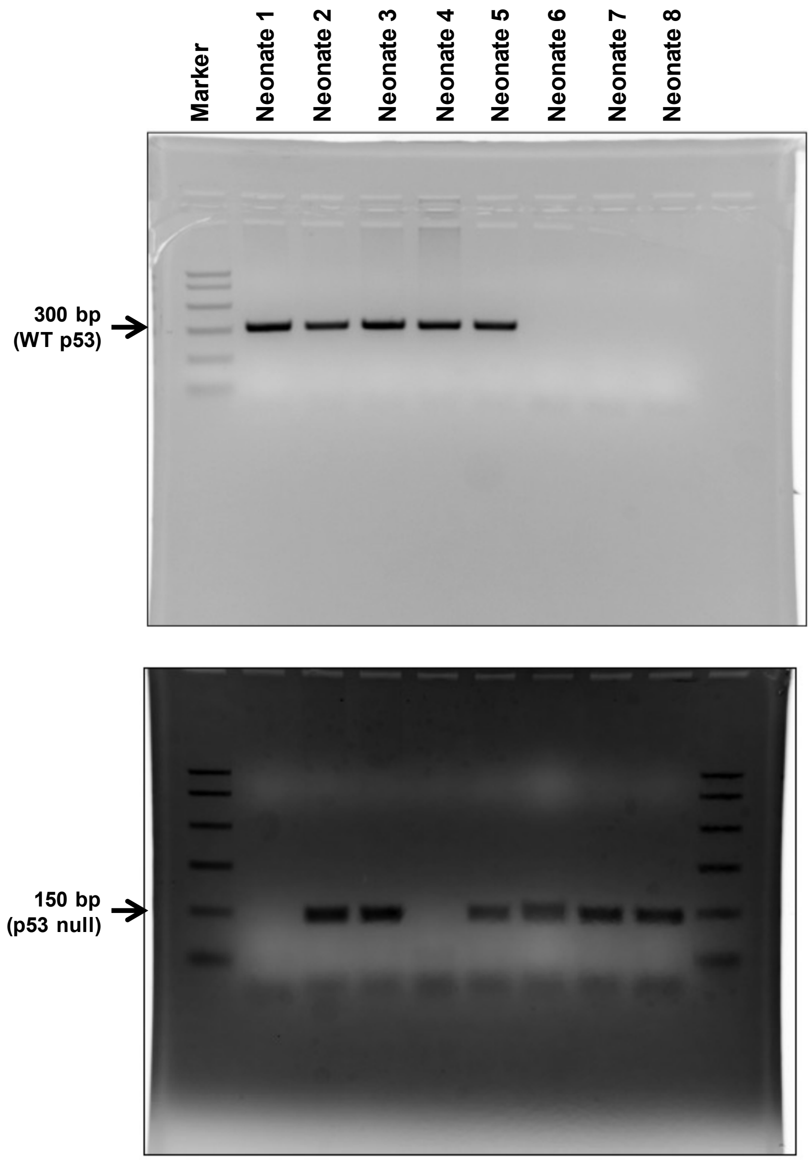

Figure S3. Images of full-length gels for Fig. S1A.

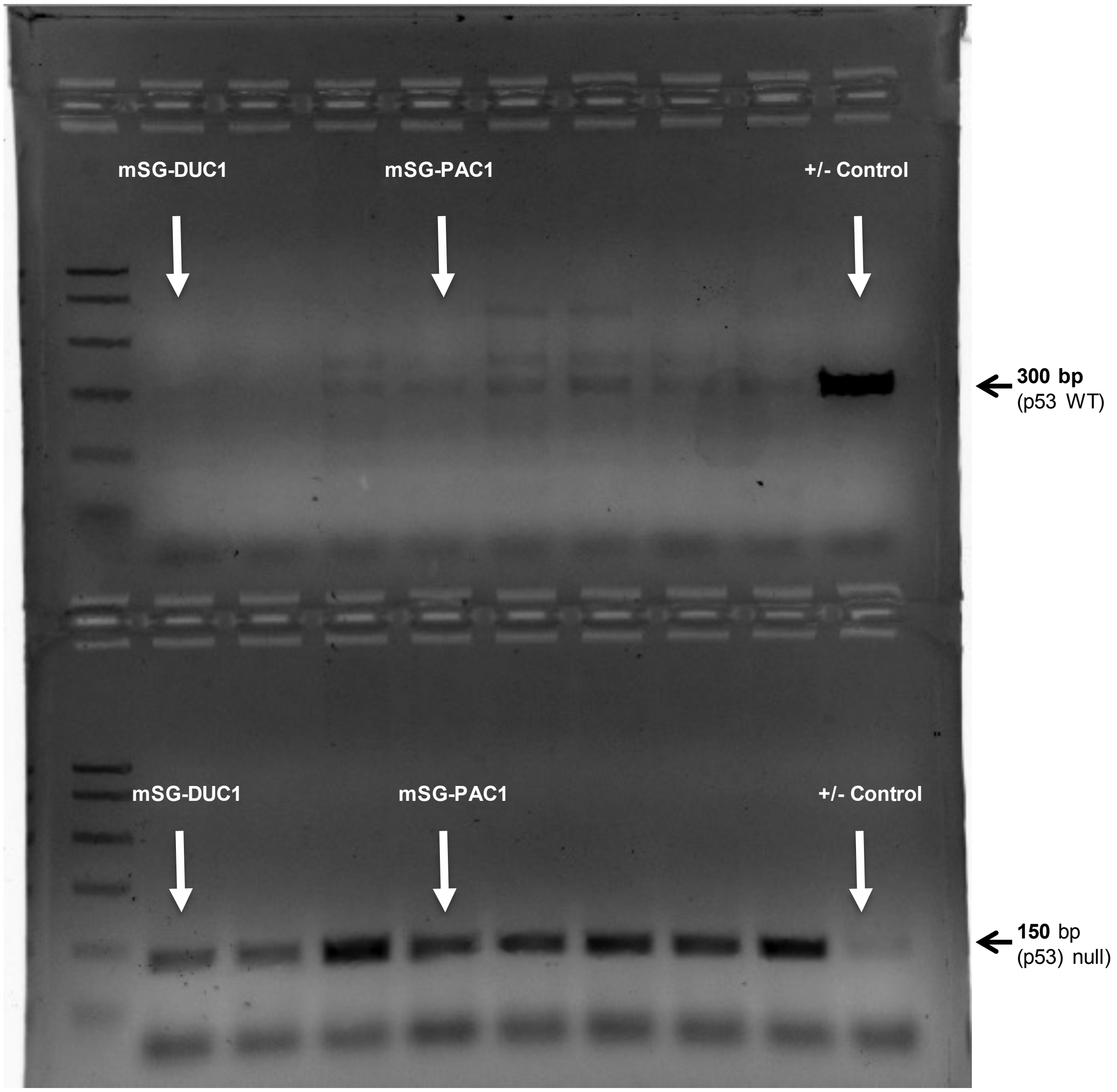

Figure S4. Images of full-length gels for Fig. S1B. The lanes representing cell lines used in this study are indicated by arrows and labeled.

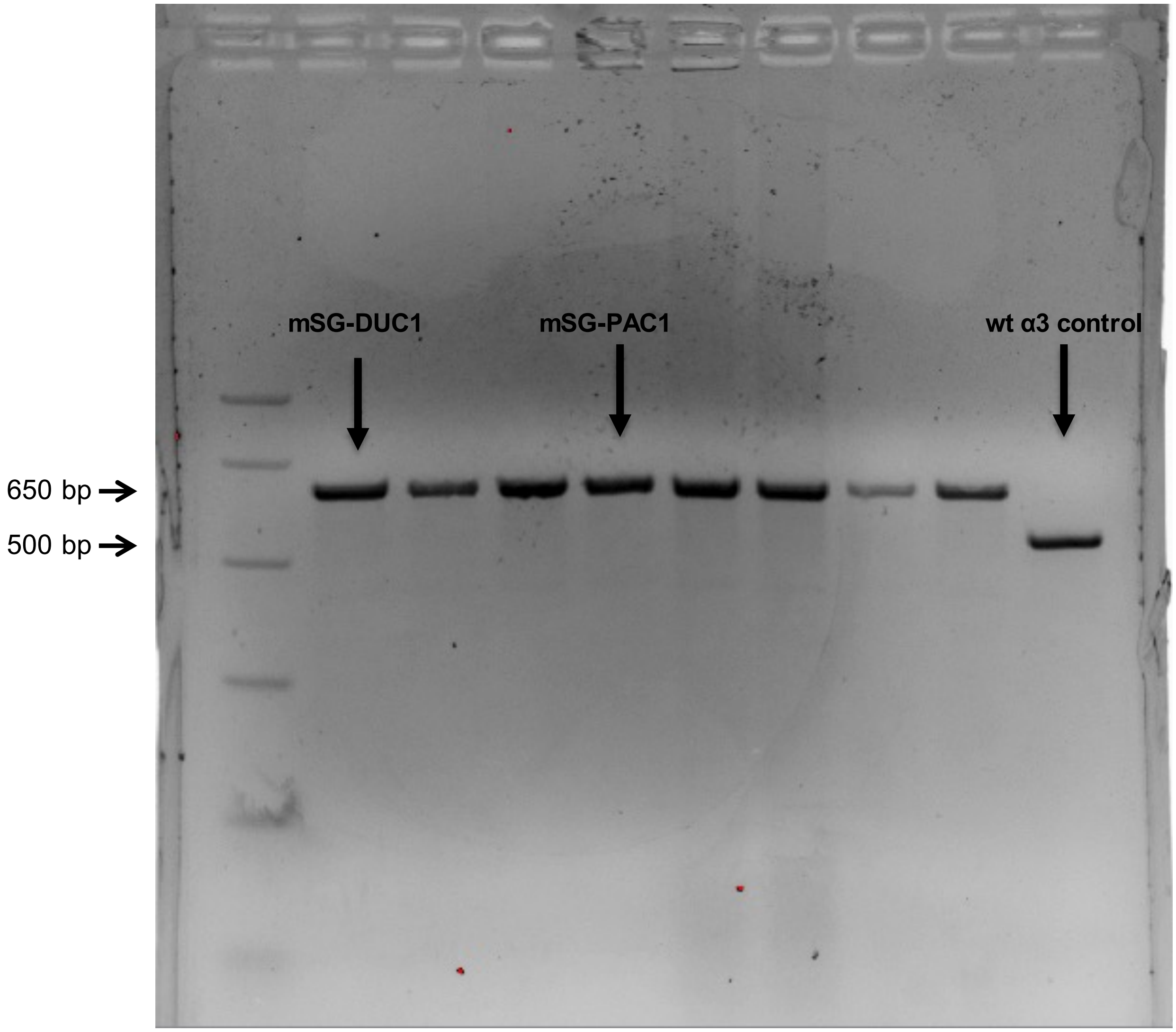

Figure S5. Images of full-length gels for Fig. S1C. The lanes representing cell lines used in this study are indicated by arrows and labeled.

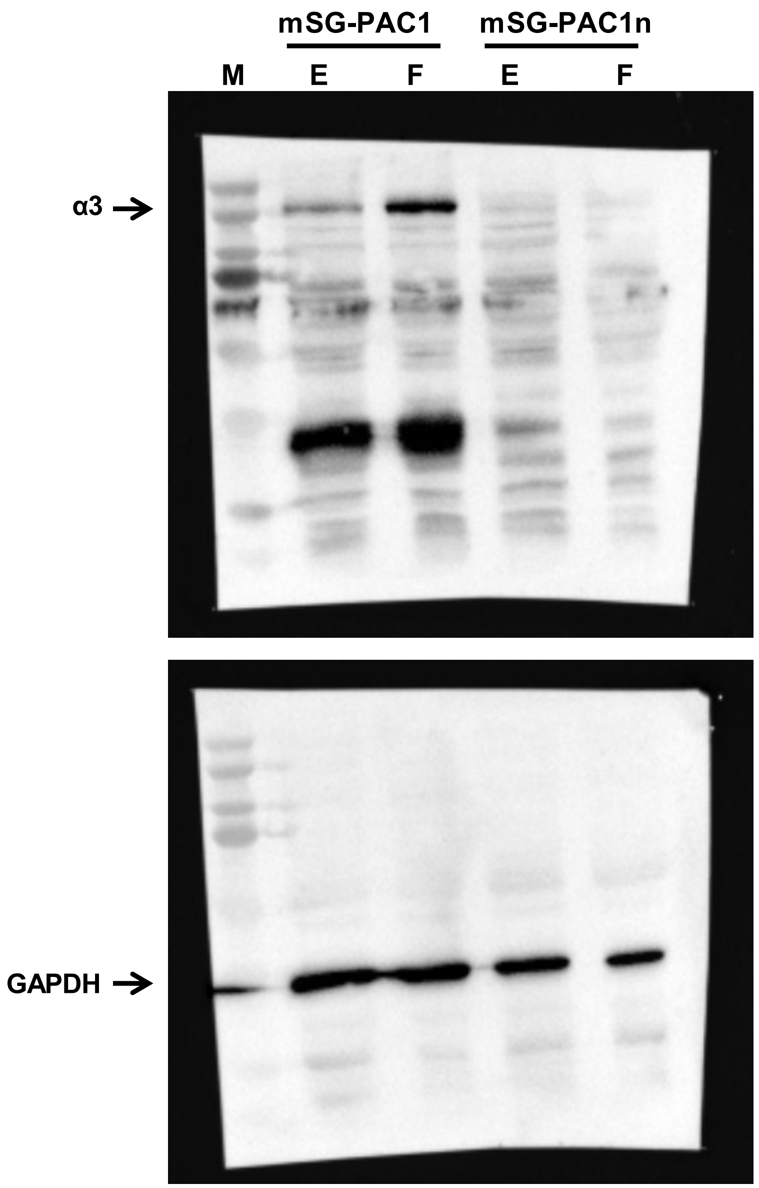

Figure S6. Images of full-length blots for Fig. 5C.

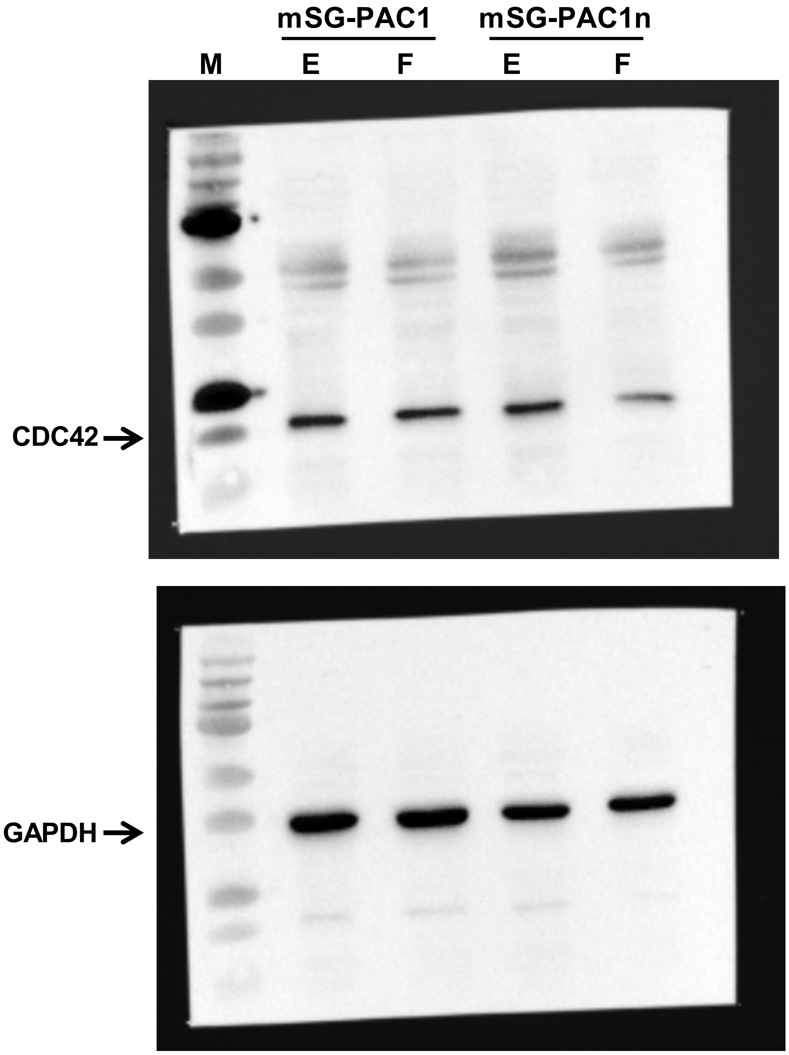

Figure S7. Images of full-length blots for Fig. 6C.

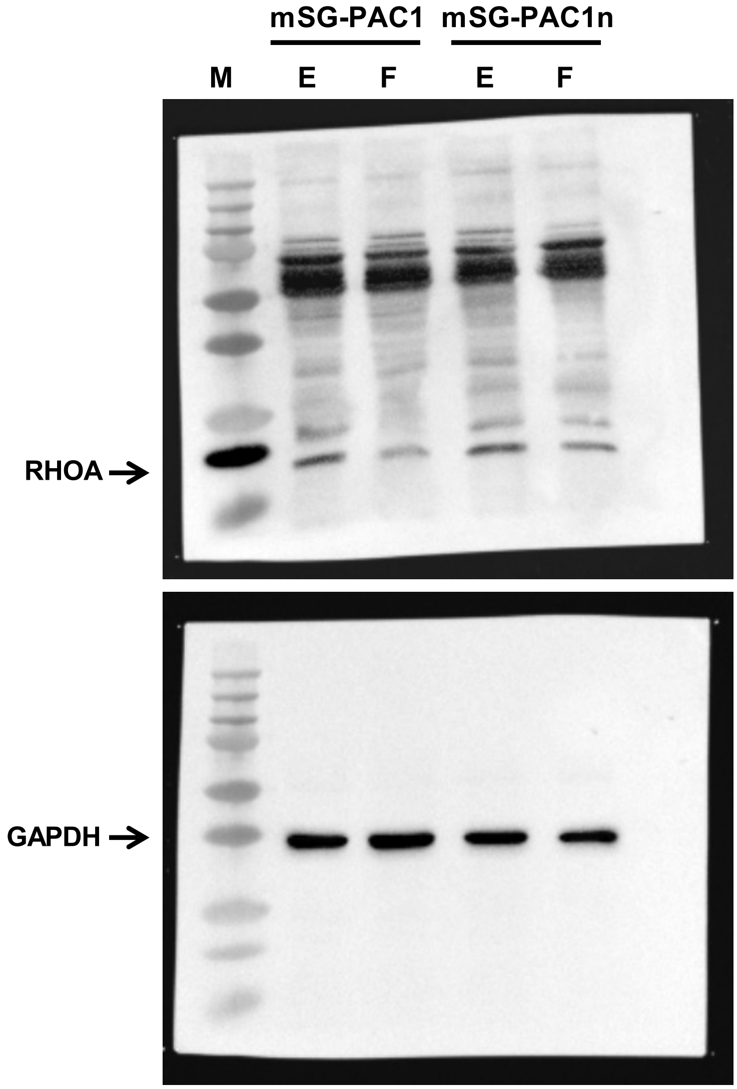

Figure S8. Images of full-length blots for Fig. 6D.
